# Supplementary material for: Inhibitory Effect of Berberine on Broiler P-glycoprotein Expression and Function: In Situ and In Vitro Studies
Source: Int J Mol Sci. 2019 Apr 22;20(8):1966. doi: 10.3390/ijms20081966 (PMC6515058; doi:10.3390/ijms20081966)
Supplement: Supplementary file 1 [file ijms-20-01966-s001.pdf]

Supplementary Material

Article title： Inhibitory effect of berberine on chicken P-glycoprotein expression and function: *in situ* and *in vitro* studies

Journal name： International Journal of Molecular Sciences

Author names： Yujuan Zhang, Li Guo，Jinhu Huang, Yong Sun, Fang He, Mire Zloh*, Liping Wang*

***Co-corresponding authors:**

Dr. Liping Wang, College of Veterinary Medicine, Nanjing Agricultural University, Nanjing, Jiangsu Province, 210095, PR China. Email: wlp71@163.com

Dr. Mire Zloh, Faculty of Pharmacy, University Business Academy, Trg mladenaca 5, 21000 Novi Sad, Serbia. Email: zloh@live.co.uk

Figure S1.

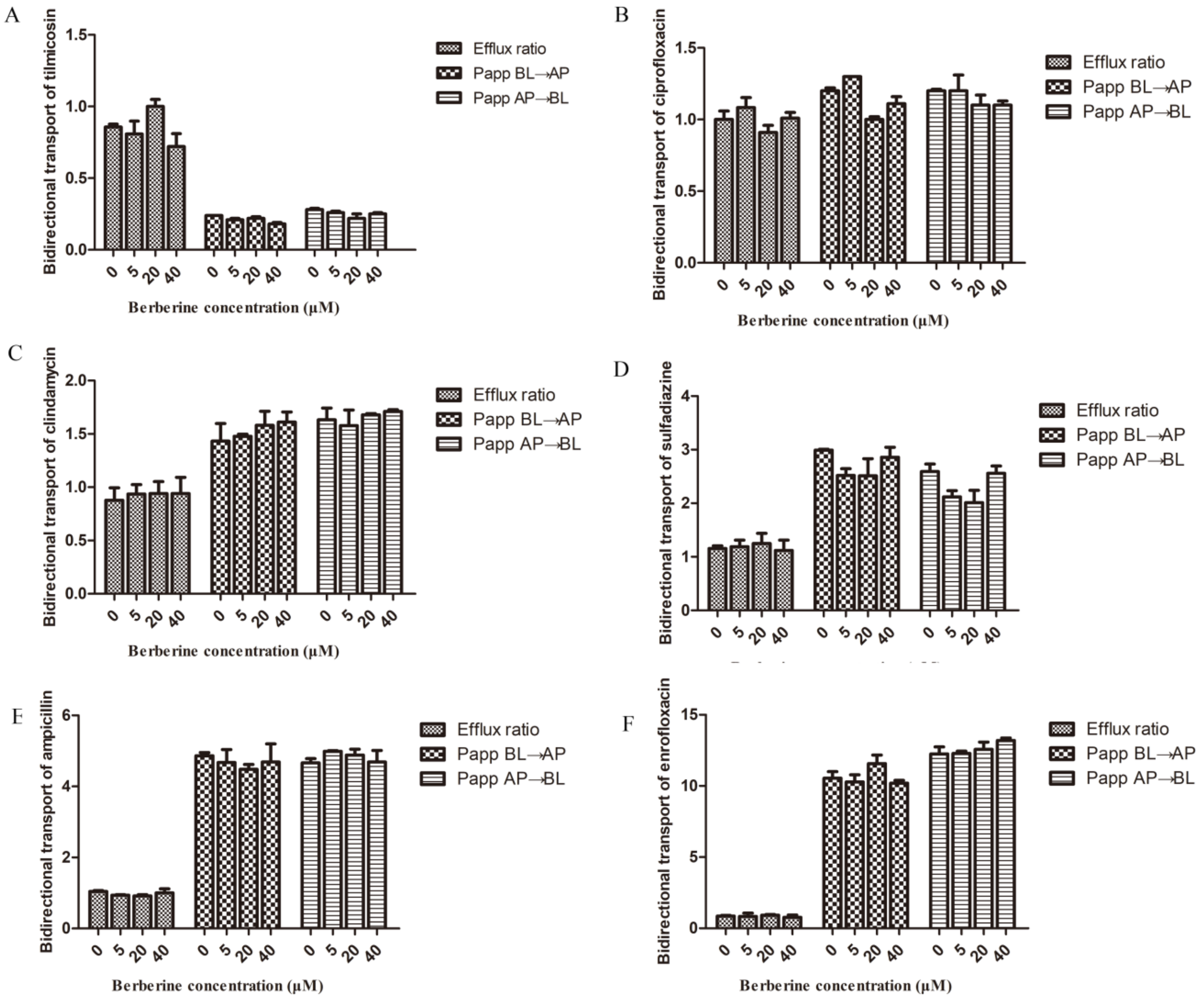

Fig. S1 Bi-directional transport of difference chicken P-gp substrates across MDCK cell monolayer after pre-treatment with berberine for 2 h. **(A)** Tilmicosin. **(B)** Ciprofloxacin. **(C)** Clindamycin. **(D)** Sulfadiazine. **(E)** Ampicillin. **(F)** Enrofloxacin. Data are shown as mean ± SEM of three independent experiments.

Table S1. Sequence alignment of mouse and chicken p-glycoprotein primary sequences based on the structural alignment of the two proteins using experimentally determined binding site of the mouse p-glycoprotein xray crystallography structure. The overlay of the two structures was achieved using structural alignment of protein structures tool implemented in Maestro. The letters in the bold and encompassed by a red frame are identified as binding site residues.

```
                                                     ..........+.........+.........+.........
3G60                                       SSA 1   57 HHHHHHHHHHHHHHHHHHHH-CC-CCHHHHHHCC-CCCCH
Homology model of p-glycoprotein in chicken SSA 2   69 CC-CCCHHHHHHHHCHHHHHCCCCCCCC-CCCCCCCCC-H
3G60                                       1       57 AIIHGVALPLMMLIFGDMTD-FA-EYYYTGIGVL-AQGMF
Homology model of p-glycoprotein in chicken 2       69 AA-HGTSLPIAMIIFGDMTDSFVLEMYA-YYSIAVAQF-Q

                                                     ..........+.........+.........+.........
3G60                                       SSA 1  302 HHHHHHHHHHH-C-CCC-HHHHHHHHHHHHHHHHHHHHHC
Homology model of p-glycoprotein in chicken SSA 2  313 HHHHHHHHHHHHHCHHHHHHHHHHCHHHHHHHHHHHHH-C
3G60                                       1      302 IYASYALAFWY-T-IGQ-VLTVFFSVLIGAFSVGQASPNA
Homology model of p-glycoprotein in chicken 2      313 LIYASYALAFWYGISIGNVLTVFFSVLIGAFSIGQTAP-A

                                                     ..........+.........+.........+.........
3G60                                       SSA 1  715 HHHHHHHHHHHHHHHHHHHHHHC---CCHHHHHHHHHHHH
Homology model of p-glycoprotein in chicken SSA 2  728 HHHHHHCCCHHHH-H---HHHHHCCCC-HHHHHHHH-HHH
3G60                                           1  715 IINGGLQPAFSVIFSKVVGVFTN---QNNLFSLLFLILGI
Homology model of p-glycoprotein in chicken     2  728 IVNGALQPAFSVI-F---SEIIGIFSS-LYSLLFLA-LGI

                                                     ..........+.........+.........+.........
3G60                                       SSA 1  968 -------------------------------HHHHHHHH-
Homology model of p-glycoprotein in chicken SSA 2  980 -------------------------------HHHHHHHHH
3G60                                           1  968 -------------------------------ENVLLVFS-
Homology model of p-glycoprotein in chicken     2  980 -------------------------------KTVFLVFSA

                                                     ..........+.........+.........+.........
3G60                                       SSA 1  976 HHHHHHHHHHHHHCCCC
Homology model of p-glycoprotein in chicken SSA 2  989 HHHHHHHHHHHHHHH--
3G60                                           1  976 AIVFGAMAVGQVSSFAD
Homology model of p-glycoprotein in chicken     2  989 VVFGAMALGQTSSFA--
```

Alignment RMSD between two binding sites: 2.442 Angstrom

Table S2. Binding site residues identified in x-ray crystallography model of mouse p-glycoprotein (PDB ID:3G60) and equivalent residues in putative binding site of the homology model of chicken p-glycoprotein. Structural alignment of protein structures tool implemented in Maestro was used to determine the putative binding site in the homology model.

PDB ID: 3G60	Homology model
M68	M80
F71	F83
Y303	I314(F315)
F332	F344
L335	L347
I336	I348
F339	F351
Q721	Q734
F724	F737
S725	S738
F728	F741
L971	F983(L984)
F974	F986
S975	S987
V978	F991(V990)

Residues in the brackets indicate neighbouring residues that are the same type as the residues in the crystal structure.

Figure S2. Overlay of berberine docking pose (thick gray lines)in the chicken p-glycoprotein binding site and the A) p-glycoprotein substrates (thin lines with morphine carbon atoms coloured in green and rhodamine 123 carbon atoms coloured in cyan) and B) p-glycoprotein inhibitors (thin lines with indinavir carbon atoms coloured in green, ritonavir carbon atoms shown in cyan and saquinavir carbon atoms coloured in purple). The protein model was obtained using homology modelling and the docking poses were obtained using Autodock Vina.

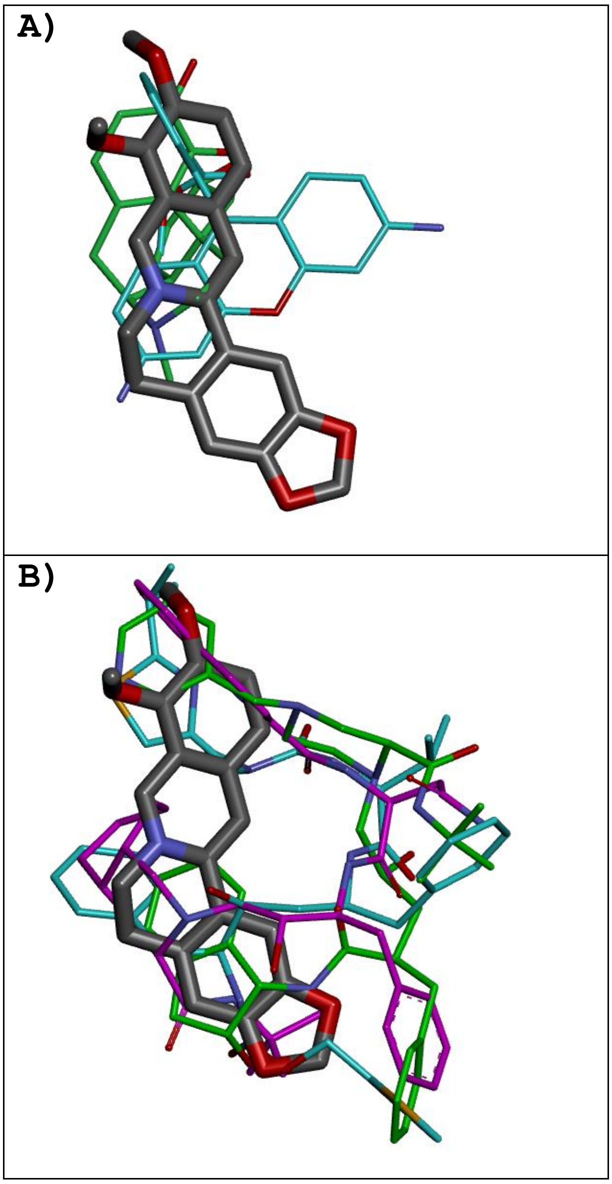

Table S3. The docking scores of berberine compared to selected P-gp substrates and inhibitors obtained by predicting binding affinities to the chicken ATP-binding cassette sub-family C, member 7 (CABCC7) and chicken multidrug and toxic compound extrusion (CMATE) transporters.

Name	Docking score (kcal/mol)[&]	
	CABCC7	CMATE
berberine	-7.9	-7.9
morphine	-6.8	-7.1
rhodamine123	-7.3	-7.9
indinavir	-8.7	-8.6
ritonavir	-7.7	-7.4
saquinavir	-7.8	-9.3

&- docking score obtained using Autodock Vina for all small molecules

Figure S3

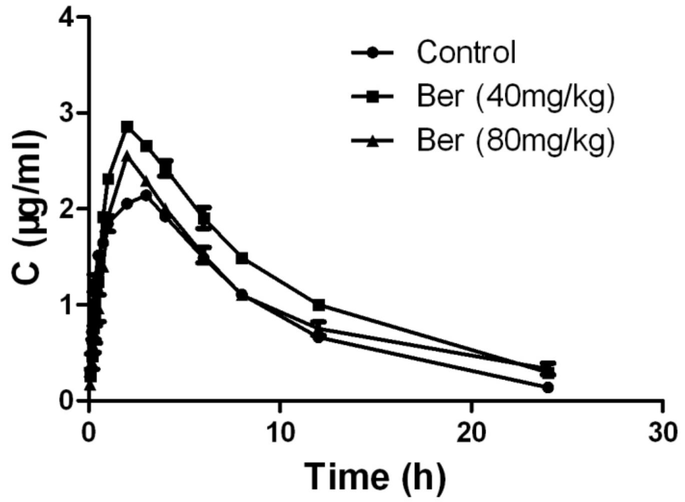

Fig. S3 Plasma concentration-time profiles of enrofloxacin after oral administration without or with different does berberine (40 mg/kg and 80 mg/kg). Data represent mean ± S.E.M (n = 5).
